# To be active through indoor-climbing: an exploratory feasibility study in a group of children with cerebral palsy and typically developing children

**DOI:** 10.1186/s12883-017-0889-z

**Published:** 2017-06-15

**Authors:** Mark Schram Christensen, Thor Jensen, Camilla B. Voigt, Jens Bo Nielsen, Jakob Lorentzen

**Affiliations:** 1 Center for Neuroscience, Section for Integrative Neuroscience, University of Copenhagen, Panum Institute, Building 33.3, Nørre Allé 20, DK-2200 Copenhagen N, Denmark; 2 Elsass Instituttet, Holmegårdsvej 28, DK-2920 Charlottenlund, Denmark; 3 DTU Compute, Department of Applied Mathematics and Computer Science, Technical University of Denmark, Richard Petersens Plads, Building 324, DK-2800 Kgs. Lyngby, Denmark

**Keywords:** Cerebral Palsy, Children, Climbing, Motor skills, peer socialization

## Abstract

**Background:**

Cerebral Palsy (CP) is the most common cause of motor disabilities in children and young adults and it is also often associated with cognitive and physiological challenges. Climbing requires a multifaceted repertoire of movements, participants at all levels of expertise may be challenged functionally and cognitively, making climbing of great potential interest in (re)habilitation settings. However, until now only few research projects have investigated the feasibility of climbing as a potential activity for heightening physical activity in children with CP and the possible beneficial effects of climbing activities in populations with functional and/or cognitive challenges. The aim of this study was therefore to test the feasibility of an intensive 3 weeks indoor-climbing training program in children with CP and typically developing (TD) peers. In addition we evaluated possible functional and cognitive benefits of 3 weeks of intensive climbing training in 11 children with cerebral palsy (CP) aged 11–13 years and six of their TD peers.

**Method:**

The study was designed as a feasibility and interventional study. We evaluated the amount of time spent being physically active during the 9 indoor-climbing training sessions, and climbing abilities were measured. The participants were tested in a series of physiological, psychological and cognitive tests: two times prior to and one time following the training in order to explore possible effects of the intervention.

**Results:**

The children accomplished the training goal of a total of nine sessions within the 3-week training period. The time of physical activity during a 2:30 h climbing session, was comparably high in the group of children with CP and the TD children. The children with CP were physically active on average for almost 16 h in total during the 3 weeks. Both groups of participants improved their climbing abilities, the children with CP managed to climb a larger proportion of the tested climbing route at the end of training and the TD group climbed faster. For the children with CP this was accompanied by significant improvements in the Sit-to-stand test (*p* < 0.01), increased rate of force development in the least affected hand during an explosive pinch test and increased muscular-muscular coherence during a pinch precision test (*p* < 0.05). We found no improvements in maximal hand or finger strength and no changes in cognitive abilities or psychological well-being in any of the groups.

**Conclusions:**

These findings show that it is possible to use climbing as means to make children with CP physically active. The improved motor abilities obtained through the training is likely reflected by increased synchronization between cortex and muscles, which results in a more efficient motor unit recruitment that may be transferred to daily functional abilities.

**Trial registration:**

ISRCTN18006574; day of registration: 09/05/2017; the trial is registered retrospectively

**Electronic supplementary material:**

The online version of this article (doi:10.1186/s12883-017-0889-z) contains supplementary material, which is available to authorized users.

## Background

Cerebral palsy (CP) is the most common cause of motor disabilities in children and young adults and it is also often associated with cognitive and psychological challenges [1, 2]. As a consequence, children with CP show less participation in social activities than their typically developed (TD) peers [3, 4]. This low early socialization is also predictive of social isolation later in life [5], which emphasizes the necessity of facilitating the motor and cognitive development of the children as early as possible. Besides individual physical therapy, children with CP should have the possibility to take part in leisure sports activities in groups of children with and without disabilities.

Performing intensive sports activities may facilitate the motor and cognitive development of the children and ensure a strengthening of their social integration, and should therefore be made available for children with CP.

Climbing involves strength, endurance [6], postural stability [7], technique, balance, coordination [8], route finding [9, 10] and attention [11], as well as a number of psychological aspects beyond fear [12], which put high demands on the participant. Independent of the participant’s level of expertise, all of the above mentioned physical and psychological abilities can continuously be challenged in climbing, because there are infinite possibilities of increasing the physical demands and challenge the participants. This is possible in climbing both on artificial climbing walls as well as during outdoor rock climbing.

Because climbing on artificial climbing walls at the same time can be considered safe in terms of number of injuries pr. 1000 h of activity [13, 14] and be mentally and physically challenging, we consider the activity ideal as a challenging way of training mental and physical abilities at the same time. Artificial climbing walls provide several possibilities for varied activities: lead sports climbing, top robe climbing and bouldering. Lead sports climbing is a safe way of climbing, if the necessary precautions are taken, where the participant clicks the safety rope into carabiners while climbing, but it provides the possibility of falling into free air for several meters before the robe catches the climber, which can give rise to mentally challenging situations [15], but may depend on climbing ability level. Top rope climbing is a very safe activity, where the safety rope always go through a safety anchor above the climbing wall. Here the focus can be on the difficulty of the movement rather than fear of falling. Bouldering, where climbing is performed above mattresses to a maximum height of 2.5–3 m without a safety rope, provides an easy way to train specific movements without the need of time consuming rope work, but requires some skills in falling appropriately. The therapeutic benefits of climbing has been studied in patients with physical disabilites [16, 17], mental disorders [18] and neurological disorders [19], where improvements after climbing was found to a similar degree or even higher than conventional therapy, but there may exist possible confounds in many of the studies of the potential therapeutic benefits of climbing (see Buechter &Fechtelpeter 2011 [20]). Studies of effects of climbing in children and adolescents [21, 22], has shown improvements in self-efficacy [22], increases in motor activity [23] and improvements in grip strength and upper body endurance, among other parameters [21]. Despite the possible benefits of climbing injuries have been observed in particular in upper extremities such as shoulders and fingers [14], but with proper warm up and precautions made to minimize certain types of grips [24] reductions in injury rates is expected.

Climbing, can be a sports activity for almost every child, including children with physical disability and cognitive deficit. For children with severe physical disability specific climbing platforms has been developed (see for instance http://www.ep-uk.com/configurations/disabled_climbing.html). The children had to understand the intention of moving against gravity by climbing, and the fact of falling by losing the grips. Fear of high, severe joint deformities, acute inflammatory joint diseases or increased risk for bone fractures needed further evaluation if climbing is an optimal sport for these children.

One of the main challenges in modern western societies is the lack of physical activity among children and in particular children with physical disabilities. Not only are children with physical disabilities less mobile, which makes it more difficult for them to participate in sports activities in general, they may also experience social stigmatization making it less likely that non-disabled children are going to engage in play and sports with them [5]. Climbing is considered an activity where the participants show a high degree of endogenous motivation to participate [25], which also means that competition between participants become less important compared with for instance team ball sports [25]. This makes climbing an activity where the individual level of expertise becomes less important, and where the individual’s relative progress becomes more important. The possibility of adjusting difficulty so it matches the individual’s level of expertise combined with both the natural pauses in-between climbing ascents and the obvious visible progress of gripping one more hold or reaching the top, makes climbing a very social sport, where participants can exchange knowledge on how to improve. Furthermore, because of the diversity of ways of climbing, training can be focused on very different aspects of physical skills, such as balance, strength, agility, flexibility, endurance, fear, attention and decision making. This makes it possible to individualize training and target specific needs, such as improving strength in a paretic arm, all within the same settings in a climbing gym, with few adjustments of climbing holds. Henceforth, we have considered climbing an ideal activity where physical disabilities may become less disabling for maintaining interaction with TD peers.

In this study, we investigated the feasibility of climbing training as a mean to activate children with CP and we further explored possible functional, physiological, cognitive and psychological changes following 3 weeks of intensive climbing training in children with CP and a group of TD. We anticipated that the climbing gym, together with skilled instructors, in combination with the notion of climbing as an endogenously motivated activity [25] in 3 weeks of intensive climbing training would I) facilitate a high level of activity among children with CP., II) improve hand coordination and increased pinch grip strength.,III) improve cognitive skills related to spatial working memory and, IV) improve the participant’s own evaluation of their physical abilities as well as skills in general. In order to test these predictions and further investigate possible changes caused by 3 weeks of climbing, we combined a set of physiological, functional, cognitive and psychological test to explore possible changes within the respective domains and shed light on possible physiological mechanisms that may change after intensive climbing training.

## Methods

### Participants

Seventeen children from the age of 11 to 13 years with (*N* = 11) and without (*N* = 6) cerebral palsy were recruited to this non randomized, non-blinded feasibility and intervention study. The children with CP were recruited according to their CP diagnosis regardless of CP type or functional level. The only exclusion criteria was severe functional dysfunction that made climbing impossible. Four of the participants knew each other prior to project (TD + CP, CP + CP). Seven of the children with CP attended mainstream school and 4 attended a school with a special program for children with physical and cognitive disabilities. All children in the TD group attended normal school.

The parents gave written and informed consent before the children participated in the study. The study was approved by the local ethics committee of the capital region of Copenhagen (H-B-2009-017). The study was performed in accordance with the Helsinki Declaration.

The participants were recruited through online advertisements. All the participants were unfamiliar with climbing training as an organized sport, a few of the participants had tried an indoor climbing wall before, but none were regular users.

Table 1 provides an overview of the participants including a note on the group that they were assigned to, based on the medical evaluation performed.

**Table 1:** Overview of the participants

Participant	Age (y)	Sex	Height (cm)	Weight^a^ (kg)	Group	Electrophysiologically Tested hand^b^	Tested leg	GMFCS
P01	11–13	F	140–150	40–50	CP	L^c^	L	1
P02	11–13	M	140–150	30–40	CP	R^c^	L	2
P03	11–13	M	150–160	40–50	CP	R^c^	R	1
P04	11–13	F	140–150	30–40	CP	R^c^	R	1
P05	11–13	M	140–150	30–40	CP	R^c^	R	1
P06	11–13	M	140–150	30–40	CP	L^c^	L	1
P07	11–13	F	130–140	30–40	CP	L^c^	L	1
P08	11–13	M	130–140	20–30	CP	L^c^	L	1
P09	11–13	M	130–140	20–30	CP	R^c^	R	1
P10	11–13	F	150–160	40–50	CP	L^c^- > R	L- > R	1
P16	11–13	M	150–160	30–40	CP	R^c^	R	1
Mean ± SD	11.6 ± 0.8	: 4 M: 7	145 ± 9.6	35.2 ± 7.5	CP (*N* = 11)		-	-
P11	11–13	F	140–150	40–50	TD	L- > R	L- > R	Ø
P12	11–13	F	150–160	50–60	TD	R	R	Ø
P13	11–13	M	150–160	40–50	TD	R	R	Ø
P14	11–13	F	140–150	30–40	TD	R	R	Ø
P15	11–13	M	140–150	40–50	TD	R	R	Ø
P17	11–13	F	170–180	60–70	TD	R	R	Ø
Mean ± SD	11.8 ± 0.9	F: 4 M: 2	153 ± 7	46.9 ± 13.2	TD(*N* = 6)	-	-	-

^a)^ Measure of weight is based on the average of three measurement performed in connection with the HUR balance test at the three test rounds. ^b)^ Based on a combined evaluation of the hand and pinch strength measurements from pre test 1 and 2 and the clinical evaluation used for sorting data for statistical analyses ^c)^Based on the participants most disabled hand for the CP group according to their own judgment, under the assumption that we would expect more room for improvement. The evaluation is then only used to sort left and right hand strength and RFD measures to measure performance changes after the climbing intervention

#### Medical evaluation

Neurological examinations were made of all participants by an experienced physiotherapist specialized in child neurology. This examination included test of reflexes, muscle tone, muscle strength, range of motion and gross motor function. History of surgery for in relation to treatments and current antispastic drug use in children with CP were recorded (see under Results).

### Intervention

The climbing facility was a climbing gym with approximately 600 m^2^ of climbing walls with walls and routes up to 12 m of height. The gym was reserved for this particular purpose during the hours that this intervention took place, which meant that the instructors and participants were the only ones present in the gym during the intervention. The gym was formerly the main climbing gym for the Danish Mountain and Climbing Club, but the club had moved to new larger (2000 m^2^) facilities. This older and smaller gym was mainly used for climbing activities with school classes. The first climbing day was performed in the larger facilities of a commercial climbing gym belonging to the Danish Mountain and Climbing Club, where the participants were provided with climbing shoes, which they used throughout the climbing period. During the first day other climbers were present in the commercial climbing gym. Nine days of climbing were planned within a period of 17 days, week 1 & 2: climbing on Monday, Tuesday and Friday, week 3: Climbing on Monday, Tuesday and Wednesday. All climbing was performed in the afternoon after school hours. Each climbing day consisted of approximately 2.5 h of physical activity. The 2.5 h were each time split into approx. 30 min of warm up exercises specifically focused on climbing. Subsequently the participants were split into two half with an approximately equal number of children with CP and TD participants in each group, where one group started with bouldering exercises, The other half of the participants were engaged in wall climbing with a top rope as safety. In order to avoid long waiting times during the top rope session, 3–4 instructors were allocated for the top rope practice, and one instructor was responsible for the bouldering session. After approximately 1 h, the two groups swapped activity. Each climbing day ended with a wrap up where the participants told what they had learned during the day. The intervention was planned as a sports activity, where the focus for the participants was to improve their climbing performance and experience that they learned new skills. Without focusing on specific needs of the individual participant with disabilities, we hoped that the focus of climbing performance also would give rise to therapeutical benefits, but intentionally without focusing on these specifically. This intentional lack of focus on therapeutical benefits was made, because we believe such a focus would remove the intrinsic enjoyment of climbing when applying external goals, see for instance [25]. The mixture of bouldering and top rope climbing provided a good foundation for a great diversity of dynamic and static movements, that challenged the participants motor skills and muscle endurance, demanding their full attention. Implementing both types of climbing also helped getting the participants to stay active and engaged longer, both physically by climbing and mentally by figuring out the routes and bouldering problems.

Three to five climbing instructors were present each day at the climbing gym during the intervention to supervise the 17 participants. The instructors were: one exercise physiologist (BSc), climbing instructor (level 3) and route setter; one neuroscientist, climbing instructor and researcher in this study, one school teacher and climbing instructor, one exercise physiologist student (M.Sc.), climber and researcher in this study, and one physiotherapist and PhD student on a project on CP and climber. The instructors did not have previous experience working with children with CP, but most of them had experience with climbing with children. The supervision consisted of constructive feedback and technical advice (e.g. active use of the legs and toes, advice on how to approach the different forms of holds such as jugs (large positive grips) and crimps (grips only held by the fingertips)) on how the participants could position their body, hands and feet in order to overcome obstacles and hard passages on the wall. Participants were also encouraged to help each other performing the different drills, and point out challenges to each other, when the instructors were not available for 1:1 coaching. During the 2 days with performance measurements (see next section) participants were cheering each other and encouraging one another to do their best. The participants were also engaged in observational learning when watching each other climbing, which have been shown to enhance motor skill learning by initiating formation of cognitive representation in memory that can be enacted and refined during overt practice, further enhancing consolidation of as much information as possible [26, 27]. Furthermore, we hoped that the vicarious experiences through observation of peers (e.g. the children with CP watching other children with CP climb) could promote a sense of personal efficacy for climbing, as it has been shown that observing or visualizing people similar to oneself perform successfully often raise efficacy beliefs in the observer [28].

To enhance social learning and strengthen group dynamics the instructors created a relaxed and playful atmosphere. It has been observed in previous studies that the feeling of relatedness (the feeling of being connected to others) enhances motivation and promote engagement in physical therapy [29–31]. With that in mind a high extent of peer socialization between the children with CP and TD group was emphasized to promote positive attitudes and acceptance instead of alienation between groups. We hoped that through positive relations the groups could inspire each other to a higher degree of task persistence and thereby facilitate the participants’ intrinsic motivation (the inherent tendency to seek out new challenges, to explore and to learn [29]) towards the climbing intervention. Furthermore, it was of great importance that the climbing instructors acted as relational key figures for the children, to establish a secure relational basis that provided the foundation for growth-orientated activity participation. Therefore, the instructors implemented and supervised games with emphasis on social interaction among the children as a fundamental part of the climbing sessions to break down physical and mental barriers by having fun.

The warm-up exercises included group- and pair-plays, where all participants were playing with each other. The exercises included chain-catch, were initially two persons had to catch the others, and when one had been captured (he/she) had to hold hands thereby acting as a unity or a chain capturing the remaining participants. The participants also had to climb on each other’s bodies and roll across a line of participants.

The climbing exercises included top rope climbing, which is an individual endeavor. However, the instructors strongly encouraged the participants to cheer each other. The instructors continuously adjusted the selection of routes to the different participants according to whether they needed a challenge (choosing a route more difficult than the instructor and participant expected was possible or a good experience (choosing an easy route to gain confidence)).

The bouldering exercises included climbing on problems defined by the instructors. The instructors tried to select problems that were just above the abilities of the participant, in order to challenge them. Often the participants were put into small groups of 3–4 participants working on the same problem, which encouraged them to share ideas on how to perform the movements. The bouldering exercises also included purely movement based tasks, like holding the hands on two pre specified holds and then trying to touch as many holds as possible with the feet. The instructors also designs exercises where specific aspects of climbing techniques were challenged, such as focus on foot-placement on small holds and holding on to edges in various orientations.

Each climbing session was also finished with all participants sitting together and telling what they had learned during the session, and explaining whether they had been able to climb better than expected.

### Test procedures

All the physiological, cognitive and psychological tests were performed at the Elsass Institute by an experienced physiotherapist specialized in child neurology or one of the two exercise physiologists that performed all assessments. The 17 participants were tested three times in all of the mentioned tests, unless otherwise stated. Two test rounds were performed prior to the intervention separated by approximately 14 days, and the 17 participants performed each test round within a period of 4–5 days on normal working/school days. The third test round was conducted the following week immediately after the last day of intervention. All the participants attended school and testing was performed such that it fitted within the participant’s normal week schedules.

Each test day lasted approximately 3 h in total for each participant. During each test day the order of tests were mixed.

#### Performance measurements

On day 3 and day 8 each participant was video-filmed on one climbing route using top rope as safety. The participants were filmed on the same route on day 3 and 8 in order to monitor progress in their climbing abilities. A route was selected by the instructors for each participant, which was judged to be just above the individual participant’s current (on day 3) climbing abilities, based on subjective evaluations by the instructors from the first 2 days of climbing. The routes were selected among 4 different routes in the climbing gym and were either 12 m or 7 m from ground level to the anchor (top point of the route) ranging in difficulty from grade 4a to 5c + (French sport climbing grading system (https://en.wikipedia.org/wiki/Grade_(climbing))). The video was subsequently used for quantification of climbing performance as measured by 1) total time on the route (from time when both feet were off the ground until the participant was holding the last hold on the wall (before he/she was lowered down)), 2) height of route climbed before decision to stop (and lowered down), 3) number of breaks (i.e. When weight was carried by the safety rope), 4) number of erroneous holds being body weight baring used by the hands, and 5) number of erroneous holds being body weight baring used by the feet.

Climbing speed (m/min) was calculated based on the total time (1) on the route and the total height climbed on the wall (2). Climbing success was calculated as fraction of route climbed. Based on the video recording the high point of climbing (2) was divided by the total height of the route (7 or 12 m). Two participants (TDs) were unfortunately not present during the last climbing days due to illness and therefore no video was recorded of them at day 8 (see also Compliance, below). Therefore performance measurements from these two participants are omitted.

On one of the 9 climbing days the children wore an electronic activity measurement device (SenseWear armband, Temple Healthcare pty Ltd., Bowral, New South wales Australia). The device estimates, based on recordings from accelerometers, thermometers and galvanic skin responses, the amount of activity performed by the wearer of the armband. Because each of the climbing days consisted of the same types of activities, the measurement of the individual participant’s activity from the 1 day they carried the armband, was used as a best estimate of their activity level during the climbing days. In order to calculate the total time that the participants in each group on average performed physical activity, we multiplied the time the individual performed physical activity with the number of days they were present for the climbing training. In order to calculate the group average, the total times were added and divided by the number of participants in each group.

During the three test sessions we obtained subjective reports of the amount of activity made during the last week from each participant using a modified version of the International Physical Activity Questionnaire (IPAQ).

### Physiological test

#### Pinch precision task

Electromyographic (EMG) activity of the hand musculature was recorded from 2 channels. Pairs of non-polarized bipolar electrodes (diameter 0.5 cm; Blue sensor, Ambu, Ølstykke, Denmark) placed on the hand over the first dorsal interosseus m. (FDI) and abductor pollicis brevis m. (APB) (interelectrode distance 1.0 cm). Before placement of the electrodes, thorough abrasion of the skin was performed to ensure the best possible signal transduction. The EMG signal were amplified (HiZx10), filtered (band pass, 5 Hz to 1 kHz), sampled at 2000 Hz and stored on a PC for off-line analysis (CED 1401,+ with Signal 5.09 software, Cambridge Electronic Design, Cambridge, UK).

To reduce electrical noise of the EMG recordings of the hand muscles a ground electrode was mounted at the distal end of ulnaris (styloid process). To eliminate electrical inferences (50 Hz noise) two Humbugs (a humbug to filtrate each EMG channel separately due to the possible different noise on the channels) were implemented in the experimental setup (Quest scientific instruments, Vancouver, Canada). Subsequent to the mounting of the electrodes the participants were instructed to perform a maximal contraction on the load cell with the thumb and index finger followed by relaxation of the hand to ensure that the EMG signal for the respective channels were of sufficient quality and stabilized over time.

The dynamic strain gauge amplifier (Dacell, Korea) connected to the load cell was adjusted to gain 800 and The EMG signal for FDI and APB muscles of the selected hand were recorded during the 90 × 7 s. frames of the visuo-motor tracking task.

The participants performed a precision grip task with the thumb and index finger where they had to make a light isometric finger pinch hold for 5 s 90 times (90 frames lasting 7 sec.) without rest with guidance from visual force feedback from the computer screen. The force level of the ramp plateau was set to 5.5 N lasting 5 s with an up-going and down-going slope lasting 0.25 s. The onset of the ramp was varied randomly between 4 different onset times. The force was measured with a load cell (gain ×800) (UU2-K30, Dacell, Korea) elevated slightly on a foam platform to place the hand in a more natural possession for performing the pinch grip. The pinch test task is depicted in Fig. 1a and b in Larsen et al. (2016) [32], with the exception that we in this study used a static pinch instead of a dynamic

**Fig. 1:**
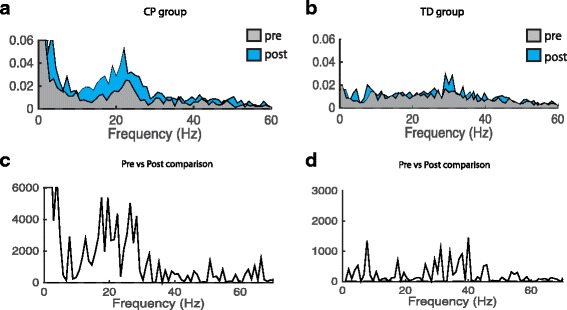
Changes in FDI_APB coherence following 3 weeks of climbing training. **a**-**d**. EMG_APB_-EMG_FDI_ coherence. Pooled Inter-muscular coherence data from EMG_APB_-EMG_FDI_ for the children with CP (*N* = 10, P16 included in the analysis, P7 excluded according to missing data) and TD (*N* = 6) group respectively. Coherence is calculated from 3.5 s hold phase in 90 trials. A and B shows pooled coherence pre (light gray) and post (light blue) climbing. **c** and **d** show the χ^2^ extended test for differences of coherence. Note that the χ^2^ values give the statistical differences between the measurements and that peak values of χ^2^ values may indicate both an increase and decrease in coherence. It is therefore not possible to determine from the bottom line of graphs which of the measurements was the largest. This can only be determined from the above graph. The dashed horizontal lines in all plots denote the 95% confidence limits χ^2^

#### Pinch strength task

Pinch strength was tested in the same setup as used for the Pinch precision task, except that force gain was set at ×200 in order to prevent saturation of the signal. Participants performed 3 maximal voluntary contractions (MVCs) with each hand using only their index finger and thumb. After countdown from 3 to 2-1-go participants were instructed to perform a finger pinch as forcefully as possible. Visual feedback of the force was provided. After the first MVC a horizontal cursor was placed indicating the maximal exerted force and was used as target (to exceed) for the next trials. At least 1 min of rest was provided between trials.

#### Pinch rate-of-force-development (RFD)

Pinch RFD was tested in the same setup as used for the precision task and strength task, except that force gain was set at ×200 in order to prevent saturation of the signal. Two vertical cursors was placed on the screen with a separation of 1.5 s. The participants were instructed to perform a brisk forceful pinch within the two vertical cursors. If the participant failed to remain relaxed before the first vertical cursor, the trial was repeated. The aim was to perform three valid trials with each hand.

#### Hand strength

The test of whole hand strength was carried out with an analog hand-dynamometer (North Coast Medical, Gilroy, California, USA). The dynamometer was adjusted to the correct hand size and the participants were instructed to hold the dynamometer with their arm adjacent to the body and elbow flexed approximately 90°. Subsequent to a short introduction and familiarization of the task, the participants performed 3 trials with each hand as forcefully as possible. The experimenter noted the force (measured in kg).

In this test both the least affected and most affected hand were tested. For the TD group this separation was done based on hand dominance.

#### Ankle contraction

Test of ankle coordination was performed on measures of EMG-EMG coherence within two recording channels. Pairs of non-polarized bipolar electrodes (diameter 0.5 cm; Blue sensor, Ambu, Ølstykke, Denmark) were placed above the TA muscle while participants performed 2 min of static ankle dorsi flexion at 10% of MVC against a constant resistance provided by the experimenter. Signals were amplified and converted into digital signals in a similar way as for the pinch task, but recorded in Spike2 software (Cambridge Electronic Design, Cambridge, UK).

#### Ankle passive stiffness

Test of ankle stiffness was performed using a hand held dynamometer (PSAD). The test was performed according to the description provided by Willerslev-Olsen et al. [33]

#### Ankle range of motion

Test of ankle ROM was performed using a hand held dynamometer (PSAD). The test was performed according to the description provided by Willerslev-Olsen et al. [33].

#### Ankle strength

Test of ankle dorsiflexion strength was performed using a hand held dynamometer (PSAD) [34]. The handheld dynamometer was during the test placed in a secure stable position while the tested person (positioned in supine position) was asked to make a dorsal flexion as forceful as possible. The maximal forced created by the dorsal flexors was recorded and taken as the maximal dorsiflexion strength.

### Functional tests

#### Sit-to-stand test

The test was made according to [35]. The participants were placed on a stool with no back-rest. Hips, knees and feet were placed in 90°. Feet were kept parallel to hip. Participants perform a test where they have to perform as many sit down-stand ups as possible in 30 s. The experimenter counted the number of times the participant sat down and stood up during the 30 s.

#### Romberg 30 s balance test

Romberg 30 s, eyes open and eye closed was employed on all participants. The participants were placed without shoes and no support on a force platform (HUR BT4 balance trainer, HUR, Helsinki, Finland). Heels were held approximately 2 cm separated with an angle of 30° between the medial sides of the feet. The arms were kept relaxed at the side of the body. Prior to the test height and weight of the participant was obtained. The outcome measure is the C90 areas measured in mm^2^, which is the area within which the center of gravity is held for 90% of the test time, as reported previously in [36]. Data from one measurement from one participant was lost due to a technical failure. One participant’s data were excluded from analysis, because the participant almost fell asleep standing with.

### Cognitive and psychological tests

#### CogState

CogState is a set of computerized tests that can be assembled from a large battery of various cognitive tests. The tests were conducted on a laptop computer in a quiet room. The instructions to each test was read aloud by the experimenter. Each test was preceded by a short training test. In this project we combined the following tests into a battery that lasted approximately 20–25 min. The tests were: **1)** a visuo-motor skill test **(CHASE)**, where the participant has to chase a moving object inside a 10 × 10 grid of locations for 30 s using clicks with a computer mouse, the outcome measure is number of correct clicks pr. sec. **2)** a test of visuo-spatial memory in the Groton Maze learning test **(MAZE)** where participants has to find a hidden path (maze) from upper left corner to lower right corner in a 10 × 10 grid of locations by revealing, using a mouse click, whether a specific location is within the correct hidden path. In the first trial participants explore the maze and in the subsequent 5 rounds they have to remember the path and move through the path as quickly as possible. **3)** a simple reaction time test, where the participants has to detect **(DETECT)** when a card on the computer screen has been flipped and press a mouse button (or keyboard). **4)** a two-choice reaction time task, where the participant can choose one of two mouse button depending on the identification **(IDENT)** of one of two colors presented on the card shown on the screen. **5)** Working memory was tested using a 1-back **(1-BACK)** task, where the participants were instructed to press one of two mouse button (or keyboard) if the presently displayed card was identical to the previously presented card. **6)** A tests of visuo-spatial learning and memory was conducted using a continuous paired association learning **(PAL)** task: Eight objects was located different places on the screen and their appearance was hidden “behind” a neutral picture, in addition three neutral pictures were distributed across the screen as well, in the center of the screen one of the eight objects was shown, and the participant had to click on the location of that specific object. Five rounds were completed; one round required that all eight objects had been correctly located. When a wrong object location was clicked, the appearance of the objects clicked was revealed. 7) Finally, a delayed recall of the Groton maze-learning test **(MAZEdelay)** was employed at the end of the test session.

Depending on which of the seven test was analyzed, speed (number of clicks pr. s.), reaction times, number of errors or accuracy, or overall normalized scores were calculated (in-build function of the software) and used for statistical tests.

#### Evaluation of personal and social competencies

In order to explore psychological changes following 3 weeks of climbing training on personal and social competencies, we employed the questionnaire “Sådan er jeg” for 4th–9th grade school children where the participants rate on a 4 point scale how they experience themselves in 72 questions [37]. The questions are ordered into five domains: Physical abilities (14 questions), skills and abilities (14 questions), mental well-being (16 questions), relation to parents (14 questions), and relation to others (14 questions). The ratings were converted into an integer scale (−2, −1, 1, 2) depending on the positive or negative content of the question and a total sum (within or across the five domains) was calculated as raw scores. The raw scores for each domain and the total scores were also transformed into stanine scores (1–9) based on a material of approximately 400 school children (Table 2).

**Table 2:** Overview of tests performed

Assessment	Outcome measurement	Comments
Physical activity		
Activity estimates	Amount of physical activity estimated as Total time with meaurement, total physcal acivity, very vigorous, vigorous, modrate, light and sedentary in [hh:mm:ss]	The SenseWear armband has been evaluted in children with relatively good results [58]
Climbing performance		
Route fraction	Fraction [0–1] of specific test climbing route climbed	Height on route is vaild method to access climbing performance and used in lead climbing competitons. We did not emoply disqialification if erroneous holds very used. Measurements a reliable to within 0.5 m, i.e. between 1/14 and 1/24 of the route fraction.
Speed	Estimate of [m/min] climbed of the test climbing route based calculated as the time from start to highest point on route incl. Breaks divided by the estiamated amount of meters climbed from ground to highest point on route.	Time on route is used in climbing competitions to separte climber reaching the same hold on a route. Our measurements were based on total tome on route incl. Breaks, and may therefore not reflect actual climbing speed, but the time spend on the route in total, we believe reflect climbing peformance to some extend. Measurements of time on route are reliable to within 1 s.
Errors (Hands / Feet)	Count [#] of number of times a hold *not* part of the test route was used either with the hands or the feet.	This measurement may not directly access climbing performance.
Functional tests		
Sit-to-stand	Count [#] of sit-stands on 30s	This test is usually done in elderly but also in children with CP to access lower body strenght. Minimum Detectable Change in children with CP is 1.8 rep/30s (based on estimates from [59] where a 5 times STS was performed).It is a reliable and valid test to measure functional strength in children with spastic diplegia [59]
Romberg	Area of sway [mm] of center of gravity in 30s with eyes open and closed	The test reflect balance abilites.
Physiological tests		
Hand strength	Whole hand pinch strength in [N]	The test acceses whole hand strength. The mesurements are reliable to within 5 N.
Pinch strength	Index-tumb finger pinch strength in [N]	The test acceses finger strength. The mesurements are highly reliable.
RFD 0–30 ms	Rate of force increase [N/s] in the first 30 ms after onset of a fast pinch	RFD measurements are the golden standard to measure explosive strength. Our equipment has high reliability.
RFD 0–50 ms	Rate of force increase [N/s] in the first 50 ms after onset of a fast pinch	Do
RFD 0–100 ms	Rate of force increase [N/s] in the first 100 ms after onset of a fast pinch	Do
RFD 0–200 ms	Rate of force increase [N/s] in the first 200 ms after onset of a fast pinch	Do
Coherence		
Finger pinch	Synchronisation of muscle activity between FDI and APB muscles measured as coherence *C(λ)*_*FDI-ABF*_ *= |f*_*FDI-ABP*_ *(λ)|* */(f*_*FDI-FDI*_ *(λ)f*_*APB-APB*_ *(λ).* Statistics is done on calculations of log transforms of areas under *C(λ)* in the interval *λ* = 15–30 Hz	The assessment partly reflect synchronisation between cortex and muscles, and is therefore an estimate of efficient of efficient motor unit recruitment [60].
Ankle Dorsiflexion	Synchronisation of TA muscle activity between two electrodes TA1 and TA2 measured as coherence *C(λ)*_*FDI-ABF*_ *= |f*_*TA1-TA2*_ *(λ)|* */(f*_*TA1-TA1*_ *(λ)f*_*TA2-TA2*_ *(λ).* Statistics is done on calculations of log transforms of areas under *C(λ)* in the interval *λ* = 15–30 Hz	The assessment partly reflect synchronisation between cortex and muscles, and is therefore an estimate of efficient of efficient motor unit recruitment [60].
Ankle joint		
Stiffness	Nm/degree	The evaluation of ROM and the ankle stiffness was performed by moving the foot from a plantar flexed position to a maximal dorsal flexed position while the participant trying to relax. The movements were made at a slow velocity (<20/s)
ROM	Range of motion in degrees	
Strength	Nm/degree	The maximal force registered during a static contraction was used to reflect strength.
Cognitive tests		
Detection	Reaction time [ms], accuracy [% correct], normalized score [AU]	The assessment tests reaction time as a basic psychomotor function. The measurements based on computer registrations are very reliable.
Identification	Reaction time [ms], accuracy [% correct], normalized score [AU]	The assessment tests choice reaction time and reflects attention. The measurements based on computer registrations are very reliable.
1-back	Reaction time [ms], accuracy [% correct], normalized reaction time [AU], normalized accuracy	The assessment reflect working memory capacities. The measurements based on computer registrations are very reliable.
Chase	Clicks pr. sec. [#]	Test the ability to use a computer mouse. The measurements based on computer registrations are very reliable.
Maze	Total number of error [#], normalized score [AU]	The assessment refect visual spatial memory and is a measurement of executive functions.The measurements based on computer registrations are very reliable.
PAL	Number of errors [#]	The assessments tests paired associative learning. The measurements based on computer registrations are very reliable.
Psychological		
Overall	Normalized scores [0–9]	The assessment is a self-evaluation test which has tested in more than 1500 school children.
Physical abilities	Normalized scores [0–9]	Do
Skills and abilities	Normalized scores [0–9]	Do
Mental well-being	Normalized scores [0–9]	Do
Relationship to parents	Normalized scores [0–9]	Do
Relationship to others	Normalized scores [0–9]	Do

### Analysis of electrophysiological data

#### EMG and coherence analysis

All surface EMG recordings of the FDI and APB muscles were analyzed offline using Matlab 2014b (Mathworks, MA, USA). Electrical recordings for one participant during pretest 2 (P7) were not obtained due to contamination of the signal of the FDI channel and therefore not available for further analysis. The coherence data were epoched in four different time intervals of 3.5 s duration during the tonic pinch grip (corresponding to the four different levels (L) of the ramp plateau; L1 = (1.5–5 s.), L2 = (1.75–5.25 s.), L3 = (2–5.5 s.), L4 = (2.25–5.75 s.)). As preprocessing steps before undertaking population analysis the epoched data were notch filtered to reduce 50 Hz noise, normalized to have unit variance, full wave rectified to maximize the information regarding timing of motor unit action potentials [38–41] and possible contamination of electrical cross-talk were removed for all EMG data by visual inspection. Only EMG amplitudes above approximately 1⁄2 of the mean EMG amplitudes were used for further analysis, in order to remove low amplitude cross talk between muscles (the Matlab application software Peakfinder was used for this purpose, http://www.mathworks.com/matlabcentral/fileexchange/25500-peakfinder-x0%2D-sel%2D-thresh%2D-extrema%2D- includeendpoints--interpolate-). Power spectra were constructed from sections of data (of 3.5 s. duration) taken at a fixed offset time with respect to trigger points in accordance with the ramp cycle levels mentioned earlier (4 cycles: [1.5–5 s.], [1.75–5.25], [2–5.5], [2.25–5.75]) [42].

Normalized signals are assumed to be realizations of stationary zero mean time series, denoted by *x* and *y*. Power spectra were constructed from sections of data taken at a fixed offset time with respect to a trigger point in each trial. Estimates of the power spectra were constructed by averaging periodograms across all trials. *F*_*xx*_ *(λ)* and *f*_*yy*_ *(λ)* represent the Fourier transforms of processes *x*, and *y*, at frequency *λ*. The cross spectrum between x and y is denoted by *f*_*xy*_ *(λ)*, and is estimated in a similar manner. Two functions were then used to characterize the signals’ correlation structure: coherence, and cumulant density. Coherence estimates are bounded measures of association defined over the range [0, 1]. The cumulant density provides an unbounded time-domain representation of the EMG-EMG correlation structure analogous to the motor unit cross-correlogram and is defined as the inverse Fourier transform of the cross-spectrum [43]. For the present data, coherence estimates provide a measure of the fraction of activity in one EMG (APB) signals that can be predicted be the activity in the other EMG (FDI) signal. In this way, the coherence in this particular experiment is an estimate for the common input to the two muscles (inter-muscular (IM) coherence). In order to summarize the correlation structure across participants, the individual coherence and cumulant density estimates were pooled providing a single time or frequency domain measure [44]. The interpretation of pooled estimates of spectrum, coherence and cumulant is similar to those obtained for individual records, except any inferences related to the population as a whole. The individual coherence and cumulant density were likewise pooled across participants to provide single time and frequency domain measures and the χ2 extended difference of coherence test were used to make statistical inferences regarding the difference of coherence between the EMG signals of the FDI and APB muscle [42]. EMG from two separate sets of TA electrodes were imported into Matlab. The procedure for analysis was the same as for the EMG^APB^-EMG^FDI^ analysis except that the data was generated during a 2 min static contraction and not a concatenation of several epochs of static contraction.

#### Analysis of pinch RFD

Force was measured using a hand held pinch device with a strain gauge providing voltage output corresponding linearly to the applied isometric force. Force onset was calculated using an initial visual inspection of the force measured. A time point prior to visible force onset was identified and a stable force baseline 500 ms prior to that point was calculated. Actual force onset was then calculated as the time point after the visually identified point that exceeded 2 times standard deviation of the 500 ms force baseline.

#### Analysis of MVC

All MVC data was exported from Signal as Matlab files and analyzed using Matlab 2014b. A customized Matlab script provided information regarding the peak values of the participants’ trials to each experimental session (pretest1, prestest 2 and posttest). The trial with the highest MVC value for the left and right hand respectively to each of the sessions was used for further analysis. The peak magnitude of the MVC data, originally measured in Volt, was converted into N.

#### Analysis of RFD

RFD data was converted into Matlab files and analyzed in Matlab 2014. A customized matlab script was designed and the RFD performed by the voluntary contraction of the thumb and index finger was calculated at different time points (30, 50, 100 and 200 ms) following onset of contraction (which was defined as the time point when the force measurement exceed 2 x SD of the baseline force level. A time point just before the force increase was set by visual inspection which was used to calculate the baseline force 500 ms before the time point set by visual inspection. When the force exceed 2 x SD of the baseline after the time point set by visual inspection, onset of the RFD task was defined). RFD for each participant (3 trials for left and right hand respectively) was first analyzed by visual inspection and all trials contaminated by activity prior to the actual contraction (resulting in upward deflection of baseline force) were discarded. The RFD for the retained trials was then obtained from the slope of the force-time curve (Δforce/Δtime) between time periods of 0-30 ms, 0-50 ms, 0-100 ms, and 0-200 ms relative to the onset of contraction, which is used to express the rate of rise in contractile force [45]. The trial with the highest RFD (N/s) for each hand was used and the mixed linear model described earlier was employed.

### Statistical tests

Analysed data from the different tests were imported into R-studios **(**RStudio Team (2015) RStudio: Integrated Development for R. RStudio, Inc., Boston, MA URL *http://www.rstudio.com* */*.**)** for further statistical analysis. A linear mixed model was used to investigate the effect of the climbing intervention on coherence by testing the specific interactions between group and intervention time and the differences between the values for pretest2 vs. the posttest in the CP and TD group. The large variability between participants was accounted for by including the ‘participant’ in the model as random effect, reparametrized interaction between group and time as fixed effects and age, height, gender as fixed effects. For all linear mixed models, Normal Quantile-Quantile plots were made, and analyses were rejected if these did not visually display an approximate linear relationship. In addition, residual plots were inspected visually in order to ensure near normal distributions.

In order to test specific hypotheses concerning the effect of the climbing intervention on the two groups we tested differences between pretest 2 and postest in the children with CP and in the TD group, and in order to test for unspecific learning effects due to multiple exposure to the same test, we tested the differences between pretest 1 and 2 in the children with CP and in the TD group.

Correlations were tested using a Pearson’s product-moment correlation.

Statistical trends ^o^) *p* < 0.1, as well as statistical significant results *) *p* < 0.05, **) *p* < 0.01, ***) *p* < 0.001 are indicated when tested.

## Results

### Participants, physical activity and compliance

Four children with CP had tendon lengthening surgery 2–6 years prior the intervention and no children received antispastic medication.

All children with CP were diagnosed with bilateral CP except one participant that was diagnosed as unilateral CP. All children with CP were ambulant and categorized with GMFCS 1 except in one case with GMFCS 2. No walking aids were used by any of the children with CP but in 6 of the children ankle orthoses were used during daytime, but not during the climbing training. No hand or elbow orthoses were used in the group of children with CP.

The neurological examination revealed generally reduced ability to produce force bilaterally in dorsal flexors, plantar flexors, elbow flexors and elbow extensors. Spasticity was identified (>/= 1 on the MAS scale) in the plantar flexors in 7 cases and elbow flexors in 3 cases. Reduced range of motion was identified (dorsal flexion <20 degrees) in the ankle joint in 6 cases whereas no reduction in elbow was found in any of the cases.

The TD children all had normal age related strength, normal range of motion in all joints-, reflex activity and walking ability.

The primary outcome of this study is that children with CP perform an equal amount of physical activity as their TD peers without having to perform training interventions that are specialized for children with physical disabilities.

Table 3 summarizes the amount of time that was spent doing physical activity during the climbing training sessions. As the results indicate, both groups wore the armband approximately the same duration (within 2 min) and performed the same amount of physical activity.

**Table 3:** Amount of time that was spent doing physical activity during the climbing training sessions

	Total duration	Physical activity	Very vigorous	Vigorous	Moderate	Light	Sedentary
All (*N* = 17)	02:32:42	**01:45:32**	00:00:04	00:14:39	01:30:49	00:46:42	00:00:28
CP (*N* = 11)	02:33:38	**01:45:27**	00:00:05	00:14:27	01:30:55	00:48:00	00:00:11
TD (*N* = 6)	02:31:00	**01:45:40**	00:00:00	00:15:00	01:30:40	00:44:20	00:01:00

Estimates of duration of physical activity based on measures from the SenseWear armband, all number indicates average duration (hh:mm:ss) of estimated physical activity. The total duration indicates how long time the participants wore the armband. All participants wore the armband at one of the climbing days

Nine of the 11 children with CP took part in all 9 climbing days, one child with CP missed 1 day due to a logistic problem, another child with CP cancelled one climbing day due to a school arrangement. Three of the TD participants took part in all climbing days and three took part in 6 of the 9 climbing days. Cancellations in the TD group were due to illness, logistics or other arrangements, not lack of interest in the training.

Using the individual’s own time spent doing physical activity, which on average was 1:45 h. We calculated the total time of physical activity performed during the climbing sessions. The children with CP performed on average 16:05 h of physical activity during the climbing specific training and the TD group performed on average 13:01 h of physical activity taking into consideration how often each individual was present at the training. The IPAQ questionnaire disclosed that all children except two (one child with CP and one TD) made the climbing training as addition to their normal activities.

The measures of the performance during the climbing intervention test climbing route is summarized in Table 3. These results show that the children with CP improved climbing performance by the fraction of the test route climbed. However, the children with CP also increased their number of erroneous hand and footholds used during the test climb. The TD group improved climbing speed.

### Adverse events

During a warm up play on one of the last days of climbing one of the TD participants accidentally sprained the wrist in a collision with one of the other participants.

### Functional tests

Significant improvement was seen after the climbing intervention in number of times the children with CP were able to perform a sit-to-stand movement (pre intervention mean 20.2 SD 4.6, median 19 IQR 17; post intervention mean 23.5 SD 5.8, median 25 IQR 18.5; *p* < 0.01). In order to investigate whether the improvement in Sit-to-Stand was related to climbing performance, we performed correlation analyses of the change in Sit-to-Stand with the change in climbing performance (Climbing speed and fraction of route climbed) from pretest 2 to posttest in the children with CP. Neither of the two correlation analyses revealed significant relationships. No significant improvements were observed in the Romberg 30 s tests. Further details can be found in the Additional file 1: S1.

### Physiological tests

#### Strength measurements

We found significant improvements in the children with CP for the pinch grip RFD calculations of the least affected hand as a result of the climbing intervention in the time intervals 0-50 ms (*p* < 0.05), 0-100 ms (*p* < 0.01) and 0-200 ms (*p* < 0.05). No significant improvements were found for whole hand-and pinch strength (MVC) for any of the groups. Further details can be found in the Additional file 1: S2 and S3.

#### Pinch coherence

Results of the EMG-EMG coherence analysis for m. FDI and m. APB are summarized in Fig. 1. We have displayed the pooled coherence for the children with CP in 1A and the TD group in 1B. Here we observed an increase in coherence for the children with CP in a spectrum of frequencies spanning ~10 - ~30 Hz when comparing pre and post intervention. This was not the case for the TD group. The χ^2^ difference is depicted in Fig. 1b for the children with CP and 1C for the TD group, showing pronounced, above the confidence interval, increase in EMG-EMG coherence during the pinch precision task. To further investigate the coherence results the difference in log normalized area under the coherence curve in the 15–30 Hz beta band was calculated and showed significant (z = 2.819, *p* = 0.0184) increases in the children with CP, when tested in a mixed linear model.

### Ankle contraction

**Fig. 2:**
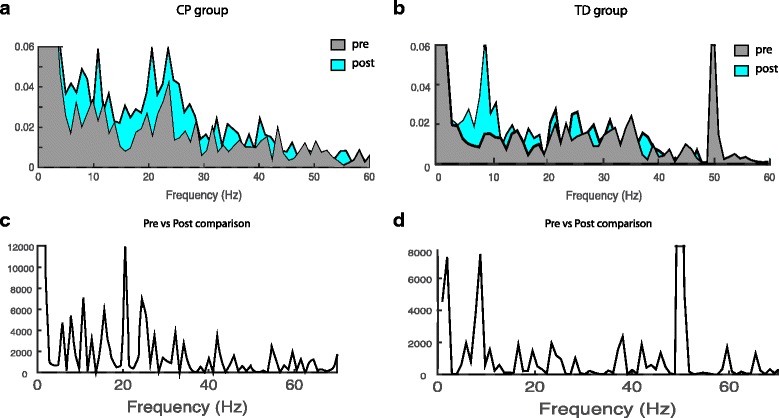
Changes in TA-TA coherence following 3 weeks of climbing training. TA-TA coherence. Pooled Intra-muscular coherence data from EMG_TA_ -EMG_TA_ pretest 2 and post climbing training for the CP (*N* = 11) group and TD group (*N* = 6) are shown in the top panel. **a** and **b** shows pooled coherence pre (light gray) and post (light blue) climbing. **c** and **d** show the χ^2^ extended test for differences of coherence and reveal the difference between the two test sessions. Test of the coherence for the TA measurements revealed modest differences in χ^2^ values in a wide frequency band for the children with CP with a peak in the 20 Hz bin

Results of the EMG-EMG coherence analysis during the 2 min of static ankle dorsi flexion are summarized in Fig. 2. We have displayed the pooled coherence for the children with CP (2A) and the TD group (2B). Comparisons of pre and post intervention in the ~10 - ~30 Hz range shows a trend towards a difference in pooled coherence for the children with CP but not for the TD group. The χ^2^ difference confirms a slight, above the confidence interval, increase in EMG-EMG coherence for the children with CP, especially around the 20 Hz bin. To further investigate the changes in coherence from pretest2 to posttest we calculated the log normalized area under the coherence curve and tested it in a mixed linear model in the 15–30 Hz beta band. This confirmed the trend (z = 1.95, *p* = 0.0994) of coherence increases in the children with CP, but did not obtain significant difference.

#### Ankle joint measurements

Unfortunately the PSAD device did not work properly on Pretest2 and we therefore only compared the measurements of ankle joint stiffness and ankle joint range of motion from Pretest1 with the Posttest. These measurements showed a significant improvement in range of motion (ROM) (*p* < 0.05) for the children with CP but not for the TD group. No significant differences were observed in ankle joint stiffness for any of the groups. Further details can be found in the Additional file 1: S4.

### Ankle strength

Ankle strength was measured as torque around the ankle joint using the PSAD. Only data from pretest 1 and posttest was acquired and compared. The linear mixed model of TA strength did not reveal a significant difference between the pretest and posttest in the children with CP or the TD group. Further details can be found in the Additional file 1: S5.

### Cognitive and psychological tests

CogState computerized tests was used to assess speed and accuracy in a detection task, and identification task (two choice reaction time task), in a 1-back working memory task, in a continuous paired association learning task and in a maze learning task.

The linear mixed model of the combined detection score did not reveal a significant difference between the pretest 2 and post test in the cardbased CogState computerized tests (the DETECT, IDENT and 1-BACK tasks) or the spatial location based tasks (the CHASE test, the MAZE test, the PAL test and the MAZEdelay test) in any of the groups. Further details can be found in the Additional file 1: S6.

### Evaluation of personal and social competencies

The results of the self-reported questionnaire “Sådan er jeg”, where the participant should self-report their experience of themselves in 72 questions in 5 different domains did not show significant differences in any of the groups when comparing pre2 and post. Further details can be found in the Additional file 1: S7 (Table 4).

**Table 4:** Summary of tests performed

	CP_pre; post_	CP _pre → post_	TD_pre;post_	TD _pre → post_
Climbing performance				
Route fraction^a^	0.6(0.23)- > 0.89(0.25)(*N* = 11)	Z = 4.77, *p* = 3.7^.^10^−6^***	0.74(0.19)- > 0.94(0.13)(*N* = 4)	Z = 1.81p = 0.127
Speed^a^ (m/min)	2.2(0.9)- > 2.9(1.0)(*N* = 11)	Z = 1.43, *p* = 0.28	3.9(1.3)- > 5.6(2.0)(*N* = 4)	Z = 2.86, *p* = 0.0084**
Errors^a^ (#) (Hands / Feet)	2.6(0–9)/4.6(0–8)- > 7.4(0–14)/8.5(0–22)(*N* = 11)	Z = 3.299, *p* = 0.0019** / Z = 2.20, *p* = 0.054^o^	0.25(0–4/0.5(0–3)- > 0.0(0)/0.0(0)(*N* = 4)	Z = −0.043, *p* = 0.999 / Z = −0.09, *p* = 0.995
Functional tests				
Sit-to-stand (#)	20.2(4.6)- > 23.5(5.8)(*N* = 11)	Z = 3.072, *p* = 0.0083**	29.0(4.5)- > 30.7(4.5)(*N* = 6)	Z = 1.044, *p* = 0.723
Romberg (open / closed)	613(550)- > 504(445)(*N* = 11)	Z = −1.35, *p* = 0.509 / z = −0.467, *p* = 0.979	408(119)- > 754(553)(*N* = 6)	Z = 0.295, *p* = 0.996 / Z = 1.917, *p* = 0.191
Physiological tests				
Hand strength (least / most)	158(58)/114(55)-> 167(51)/128(60)(*N* = 11)	Z = 1.067, *p* = 0.708 / Z = 1.715, *p* = 0.283	216(82)/195(68)-> 227(69)/201(58)(*N* = 6)	Z = 0.745, *p* = 0.895 / Z = 0.208, *p* = 0.999
Pinch strength (least / most)	34.8(6.3)/29.4(8.7)-> 37.5(7.0)/32.2(8.3)(*N* = 11)	Z = 1.165, *p* = 0.639 / Z = 1.009, *p* = 0.746	43.1(12.0)/40.9(12.2)-> 47.3(14.9)/43.1(14.7)(*N* = 6)	Z = 1.263, *p* = 0.569 / Z = 0.441, *p* = 0.983
RFD 0–30 ms (least / most)	59.2(58.6)/50.6(64.4)-> 79.6(68.6)/54.5(68.6)(*N* = 11)	Z = 1.797 *p* = 0.242 / Z = 0.714,*p* = 0.908	69.3(49.0)/41.6(13.3)-> 72.6(29.6)/54.2(25.4)(*N* = 6)	Z = 0.003, *p* = 1.0 / Z = 1.493, *p* = 0.412
RFD 0–50 ms (least / most)	79.0(63.7)/80.0(79.4)-> 109.1(76.6)/90.6(84.8)(*N* = 11)	Z = 2.317, *p* = 0.0752^o^ / Z = 1.045, *p* = 0.723	99.7(66.4)/72.1(28.0)-> 113.6(45.6)/79.6(29.3)(*N* = 6)	Z = 0.584, *p* = 0.953 / Z = 0.269, *p* = 0.997
RFD 0–100 ms (least / most)	110.9(53.1)/109.5(78.5)-> 154.4(56.5)/123.3(76.8)(*N* = 11)	Z = 2.939, *p* = 0.0174* / Z = 0.872, *p* = 0.830	147.5(87.6)/132.3(57.9)-> 171.6(70.9)/133.4(47.5)(*N* = 6)	Z = 0.989, *p* = 0.759 / Z = −0.208, *p* = 0.999
RFD 0–200 ms (least / most)	105.6(36.4)/91.9(45.4)-> 133.9(18.7)-101.4(40.5)(*N* = 11)	Z = 2.309, *p* = 0.0768^o^ / Z = 0.733, *p* = 0.900	133.2(53.9)/123.4(54.6)-> 145.4(60.3)/135.6(49.1)(*N* = 6)	Z = 0.595, *p* = 0.9498 / Z = 0.527, *p* = 0.967
Coherence				
Finger pinch (FDI-APB)	-1.73(0.84)- > −0.89(0.91)(*N* = 10- > 11)	Z = 2.81, *p* = 0.0184*	−1.58(0.63)- > −1.49(0.83)(*N* = 6)	Z = 0.239, *p* = 0.993
Ankle Dorsiflexion (TA-TA)	-0.96(0.88)- > −0.57(0.98)(*N* = 11)	Z = 1.951, *p* = 0.0994^o^	−1.10(0.45)- > −0.91(0.82)(*N* = 6)	Z = 0.644, *p* = 0.7964
Ankle joint				
Stiffness^b^	4.57(1.86) - > 4.08(1.82)(*N* = 11)	Z = −1.002, *p* = 0.533	5.05(1.80)- > 5.17(1.18)(*N* = 5)	Z = 0.379, *p* = 0.913
ROM^b^	62.5(12.0) - > 67.98(7.76) (*N* = 11)	Z = 2.764, *p* = 0.0114*	63.12(6.62)- > 66.16(5.16)(*N* = 5)	Z = 1.019, *p* = 0.5214
Strength^c^	25.9(9.5)- > 30.2(12.0)(*N* = 11)	Z = 1.387, *p* = 0.304	45.9(17.5)- > 40.7(13.8)(*N* = 6)	Z = −1.476, *p* = 0.260
Cognitive tests				
Detection^d^	83.3(10.9)- > 83.1(10.0)(*N* = 11)	z = −0.048, *p* = 1.0	98.3(8.5)- > 95.7(7.3)(*N* = 6)	Z = −0.648, *p* = 0.933
Identification^d^	87.4(8.0)- > 87.5(9.6)(*N* = 11)	Z = 0.032, *p* = 1.0	94,2(6.3)- > 95.3(6.7)(*N* = 6)	Z = 0.345, *p* = 0.993
1-back (speed/accuracy)^d^	959(139)/82.3(9.4)-> 962(214)/82.0(7.9)(*N* = 11)	Z = 0.199, *p* = 0.999 / Z = −0.190, *p* = 0.999	768(163)/91.3(12.7)-> 753(198)/91.7(9.4)(*N* = 6)	Z = 0.473, *p* = 0.978 / Z = −0.039, *p* = 1.00
Chase^e^	1.02(0.23)- > 1.15(0.23)(*N* = 11)	Z = 2.057, *p* = 0.140	1.54(0.23)- > 1.48(0.22)(*N* = 6)	Z = −0.646, *p* = 0.934
Maze^d^	99.6(6.5)- > 103.2(6.4)(*N* = 11)	Z = 1.721, *p* = 0.279	101.3(3.7)- > 105.2(5.1)(*N* = 6)	Z = 1.453, *p* = 0.438
PAL	45.6(51.1)- > 32.0(62.9)(*N* = 11)	Z = −1.940, *p* = 0.181	15.3(15.8)- > 6.5(3.4)(*N* = 6)	Z = −1.122, *p* = 0.670
Psychological				
Overall	4.00(2,05)- > 4.63(2.41)(*N* = 11)	Z = 1.327, *p* = 0.524	5.5(2.42)- > 6.50(2.07)(*N* = 6)	Z = 1.484, *p* = 0.418
Physical abilities	4.64(2.73)- > 4.45(2.73)(*N* = 11)	Z = −0.342, *p* = 0.993	5.83(2.93)- > 5.83(2.99)(*N* = 6)	Z = −0.083, *p* = 1.000
Skills and abilities	4.54(2.25)- > 4.45(2.38)(*N* = 11)	Z = −0.216, *p* = 0.999	4.33(2.5)- > 5.67(2.16)(*N* = 6)	Z = 1.896, *p* = 0.198
Mental well-being	4.09(2.21)- > 4.91(2.59)(*N* = 11)	Z = 1.457, *p* = 0.436	5.83(1.72)- > 6.67(1.96)(*N* = 6)	Z = 1.024, *p* = 0.736
Relationship to parents	3.18(1.89)- > 4.18(2.36)(*N* = 11)	Z = 1.481, *p* = 0.420	7.33(1.63)- > 6.83(1.72)(*N* = 6)	Z = −0.516, *p* = 0.970
Relationship to others	4.45(2.06)- > 5.27(2.37)(*N* = 11)	Z = 1.389, *p* = 0.481	7.67(0.82)- > 7.0(1.55)(*N* = 6)	Z = −0.804, *p* = 0.867

Summary of all tests performed and the effect of the climbing intervention. Z-scores and *p*-values are obtained from the employed linear mixed models where four tests have been made, Pre1 vs Pre 2 and Pre 2 vs Post both in the CP and TD group. Only statistics from the pre2 vs post session are presented, but *p*-values are adjusted for multiple comparisons where the pre 1vs pre2 comparison is included.

(Least/Most): indicate test of changes in the least or most affected hand. ^a^ Data from two time points only at day 3 and day 8 of the climbing intervention days. ^b^ Data from Pre1 and Post only due to technical issue with the measurement device at Pre2 test. ^c^ Data only from Pre2 and Post. ^d^ Tests for the combined score-measurements are shown here. The individual test of speed and accuracy are not presented here. None of them revealed significant differences when comparing the effect of the climbing intervention in any of the two groups. ^e^ The chase test revealed a significant improvement in number of clicks per sec. In the TD group between pre1 and pre2. ^o^) *p* < 0.1, *) *p* < 0.05, **) *p* < 0.01, ***) *p* < 0.001. Correlation analyses was performed between the changes in the Sit-to-stand, ROM, RFD 0-100 ms and Coherence between FDI and APB measurements the improvements in the climbing abilities in the children with CP. However, none of correlations were significant

## Discussion

### Summary of results

We have shown that children with CP can engage in climbing training on equal terms with their TD peers. The children with CP performed equally many hours of physical activity as their TD peers, which amount to more than 5 h per weeks of moderate or vigorous physical activity.

Furthermore, four children with CP and three of the TD children and their parents expressed a wish to continue the climbing training after the 3-weeks ended. On a side note it is also worth mentioning, that three of the children after the 3-week period continued climbing as an organized sport in a local climbing club.

In the present study we have shown that children in the age range of 11 to 13 with and without cerebral palsy can improve climbing abilities with less than 3 weeks of intense climbing training. Furthermore, we have demonstrated that some aspects of physiological measures as well as functional measures can be improved in children with CP with less than 3-weeks of climbing.

In summary, children with CP improved the fraction of the test route climbed, but at the expense of producing more hand placement mistakes while climbing. The children in the TD group did not improve the fraction of route climbed but increased their climbing speed.

With respect to functional improvements, children with CP improved their Sit-to-stand score, a measure used as a general measure of motor abilities.

The physiological tests revealed a significant increase in EMG-EMG coherence in the pinch task for the children with CP after the climbing intervention. Furthermore, the children with CP increased RFD in their least affected hand after the climbing intervention and had increased range of motion in the ankle joint.

None of the cognitive computer tests or the psychological questionnaire revealed any significant changes in either of the two groups when comparing before and after climbing training.

This study extends upon previous studies of biomechanical and physiological effects of sport climbing [7, 46–50]. Previous studies of psychological effects of rock climbing has previously focused on performance anxiety [51–53], attention [11] or the experience of flow [25] but to our knowledge no other studies has focused on effects of climbing training upon other psychological domains.

### Functional significance

We have shown that a short intense intervention period of climbing training can improve climbing performance in children with CP GMFCS level I and II. We have also shown that a very basic test of general functional performance reveals improvements after the climbing intervention. Furthermore we have shown that fine motor control of the index and thumb is changed as reflected by an increase in coherence in the most affected hand of the children with CP. In the least affected hand the children with CP improved RFD from 0 to 100 ms. Finally, we have shown that climbing training increase the ankle joint ROM in the children with CP measured on the most affected lower limb. These findings suggest that climbing training have physiological impact on both upper and lower limb physiology and that climbing training can improve aspects of general functionality as measured by the Sit-to-stand test.

The psychological as well as cognitive tests did not reveal any significant improvements. All though climbing training has mental aspects related to management of fear and anxiety and cognitive aspects related to decision making and planning we were not able to see any effects on the employed measurements. This may be due to several reasons. The number of participants was rather small compared to the groups that the psychological test (Sådan er jeg) has been evaluated on.

### Clinical significance

Climbing training seems to be a way to engage children with CP in many weekly hours of physical activity in a fun and motivating way that may help improve climbing skills, muscle strength, balance, as well as mental and social skills. Therefore, we propose climbing as an efficient clinical training method with several benefits for children with CP.

This is also what we experienced during the intervention. Although the children had very diverse physical capabilities, they all found routes that suited their different skill-sets. With guidance from the instructors the participants could progress on these routes, often in collaboration with their peers from both the CP and the TD group. The mixture of top rope and bouldering provided a good foundation for the participants to try many different aspects of climbing, and for the instructors it increased the possibilities for setting up playful climbing games where the participants could interact independently of their physical foundation.

The participants self-reliance in relation to their climbing abilities may have improved during the 3 weeks and that the immediate feedback that the nature of climbing provided (e.g. overcoming specific obstacles on the route), may have fueled the children’s pursue of their inherent enjoyment rather than external reward or influence. Some of the children even personalized the routes, as one of the children with CP expressed it: He wanted to overcome his ‘arch enemy’ (the route he progressed on for several weeks). The participants had surprisingly few complaints about the level of their physical exertion, even though the sessions were relatively long and challenged the participants both physically and mentally. This may reflect that the climbing environment and the instructors encouraged the participants to develop the right mindset and self-determination for pushing their limits. In that context, it was enjoyable to see that the children with CP met the challenge with the same enthusiasm as the healthy controls, even though the training was not specifically adapted to the children with CP. Future studies could have even more focus on customizing the climbing training in order to accommodate the specific needs of participants with disabilities. Especially it could be of interest to investigate if climbing training can be used as an effective tool to treat people with even more severe CP. It should also be noted that the clinical significance of climbing has started to be recognized in other patient groups as well (e.g. in people with multiple sclerosis and geriatric patients) [54–56].

### Comparison with previous studies

Only very few studies have been conducted looking at effect of (therapeutic) climbing training in children and adolescents [21–23]. We did not find significant changes in hand strenght. The average hand strength measurements reported by pervious studies in youth climbers at a comparable age [21] are within the range which we also observe (between 0.25 and 0.69 (kg/body weight) in the most dominant hand. However, the ranges for the study by Balas is unknown. Balas et al. used 8 weeks of training where we only look at the 3 weeks of training. That may be a reason to why we do not see changes in hand strengt. Balas only observed changes in the group that performed a high volume of climbing. Unfortunately we do not have any reliable way of estimating the amount of climbed meters as Balas et al. had.

With respect to the intervention intensity, previous studies have used climbing once or twice a week for 8 weeks [21] for children that already were climbing, A study of climbing in children with special needs Mazzoni et al. (2009) used 1 hour of climbing once a week for 6 weeks [22]. Therme et al. 1992 used 6 training session (unfortunately, only access to the abstract was available) so further details of the intervention are not possible to obtain [23]. Our intervention protocol was more densely packed, but in terms of total duration pr. day, but also in terms of number of sessions per week (three vs. one or two. However, we do not believe this relatively higher intervention intensity had a major role to play in terms how compliance. For all cases of absentee, reasons such as already planned family arrangements, missing transportation to the climbing gym and illness were given. This relatively high weekly frequency of climbing was used, because the overall duration of the project had to be limited, but future interventions would more ideally be spread over a longer total duration with max 2 training sessions per, week, with a possible gradual increase in weekly frequency.

### Perspectives

Children with CP are often confronted with the challenge that they are not disabled enough to participate in adaptive sports, but fall short in traditional competitive sports, which can lead to a loss of motivation and self-confidence [57]. Climbing as an intervention tool has the advantage that the participant competes against him- or herself and the many different routes makes it possible to adjust the level of difficulty according to the requirements of the individual. This provides a basis for successful experiences in a social environment, independent of the level of expertise. Climbers of different levels may climb together or alongside each other with the possibility of the more experienced climbers providing positive feedback to the less experienced, further enhancing good relations between climbers

One can hope that the climbing environment in the future can be an arena for children with CP where they can develop social, mental and motor skills alongside their healthy peers, without the fear of falling short of certain competitive standards. The use of climbing as a tool in physical therapy is still a largely uninvestigated area and therefore much more research should be done on the possible physical, psychological and social benefits of climbing.

### Methodological considerations

This study was designed as an exploratory study in order to investigate whether climbing training has the potential to be used as a possible therapeutic intervention, which combines physical and psychological challenges. In order to take into account possible exposure bias to the employed tests, we decided to compare two pre-intervention tests against the pre-post intervention comparison. We tested the differences between the pre1 and pre2 test and only found a significant improvement of the chase test in the CogState test battery for the TD group. All other tests did not show any significant differences between the pre1 and pre2 test. We therefore believe possible learning effect are minimal, and therefore differences between pre2 and post are more likely due to an interventional effect rather than an unspecific learning effect. Some of the employed tests, such as the questionnaire “Sådan er jeg” and the CogState tests may be very general, and not specific enough to test effects of the intervention. In particular we believe the “Sådan er jeg” questionnaire, after having employed it, is not suited for these short term intervention studies. With respect to the CogState tests we hoped to see effects on the MAZE-LEARNING test because the skills that the test resembles are similar to what you learn when trying to memorize a climbing route. The remaining tests in the CogState battery were less climbing-skill related.

The study was designed with the purpose of exploring whether children with CP can engage in climbing training together with their peers and explore possible physiological, psychological and social gains from climbing training. To explore these parameters in the most reliable way, practical issues from the surrounding activities, such as getting to the gym, organizing climbing coaches, etc. was taken care of by the experimenters and not the participant’s and their parents to the same degree as “normal” climbing training would require. Therefore, the surrounding circumstances were somewhat easier to cope with for the participants and their parents. We employed a relatively high instructor-to-participant ratio with 3–5 instructors to 17 children. This was done because we wanted to focus on both bouldering and top rope climbing, where the latter requires someone who can belay (hold the rope through a breaking device) the climber. Under normal circumstances, part of a longer climbing training course, belaying would be something that was taught the participants in the beginning or taken care of by parents. But because we wanted to focus on climbing as a physical activity (with it’s related aspects) we did not teach belaying techniques during the 3-week session. Furthermore, the activities took place in the early afternoon after school, where the parents of the children were still at work. It has to be considered that the instructors were highly motivated towards the intervention and already prepared that special precautions had to be made in order to properly adjust the climbing training to the children with CP. We therefore acknowledge that this fact potentially could bias the effect of the intervention to favor the children with CP, but when an instructor deals with a participant it would be impossible not to individualize the instruction without paying attention to the individual participant’s needs. At the same time the setting of the climbing intervention took place in a safe and inclusive environment, where the instructors facilitated interation between all participants. Therefore, in order to implement children with CP in real life climbing settings, a challenge could be the recruitment of dedicated instructors that are willing to make an extra effort, preferably with some prior knowledge of CP. However, a specialized team has been established for 6 children with CP where they climb once a week in a public climbing gym. Further, the disability level of the children with CP in this study has to be considered. The Children in our study was (with one exception) level 1 on GMFCS scale and therefore had a reasonable level of functional capability. Therefore, another consideration is how well a climbing intervention like ours could be implemented for more severely challenged children with CP.

### Statistical considerations

Despite the very small group of participants included in this study, we were able to show some changes in the functional and physiological tests. However, given the large number of tests we performed none of the tests would survive strict Bonferroni correction for multiple comparisons. We have therefore decided to show all tests uncorrected in order to give a better impression of the raw results from the individual tests. We also decided not to correct for multiple comparisons because many of the tests cannot be considered completely independent. As an example, we found high correlations between performance in the computer based chase test and most of the other computer based cognitive tests. Also, tests like the four different RFD measurements are non-independent because they include data from the same time-interval.

## Conclusion

These findings show that it is possible to use climbing as a setting for peer socialization (climbing in a mixed group of children with CP and their TD peers) as a motivating and effective training alternative in children with CP. The improved motor abilities obtained through the training is likely reflected by increased synchronization between cortex and muscles, which results in a more efficient motor unit recruitment system that may be transferred to daily functional abilities. It was not possible with this short-lasting intervention to demonstrate larger muscle strength or cognitive or psychological effects of the training.

## Additional file

Additional file 1: S1. Functional tests. These data describes the results of the Sit-to-stand- and the Romberg tests. **S2.** Whole Hand strength and pinch strength. These data describes the results of the Hand strength and Pinch strength. **S3.** Pinch rate of force development. These data describes the results of RFD 0-30 ms; RFD 0-50 ms; RFD 0-100 ms; RFD 0-200 ms for the least and most affected arm. **S4.** Ankle joint measurements. These data describes the results of the ankle joint stiffness and ROM measurements. **S5.** Ankle strength measurements. These data describes the results of the ankle strength measurements in TA. **S6.** Cognitive and Psychological test. These data describes the data of cardbased CogState computerized tests and test of the spatial location based tasks. **S7.** Evaluation of personal and social competencies. These data summarize the results of the evaluation of personal and social competencies separated in sections including Overall; Physical abilities; Skills and abilities; Mental well-being; Relation to parents; Relation to others. (DOCX 35 kb)

## Abbrevations

APB: Abductor pollicis brevis
CP: Cerebral palsy
FDI: First dorsal interosseus
MVC: Maximal voluntary contraction
RFD: Rate of force development
ROM: Range of motion
TA: Tibialis anterior
TD: Typically developed
