# Supplementary material for: To be active through indoor-climbing: an exploratory feasibility study in a group of children with cerebral palsy and typically developing children
Source: BMC Neurol. 2017 Jun 15;17:112. doi: 10.1186/s12883-017-0889-z (PMC5472985; doi:10.1186/s12883-017-0889-z)
Supplement: Additional file 1: S1. — Functional tests. These data describes the results of the Sit-to-stand- and the Romberg tests. S2. Whole Hand strength and pinch strength. These data describes the results of the Hand strength and Pinch strength. S3. Pinch rate of force development. These data describes the results of RFD 0-30 ms; RFD 0-50 ms; RFD 0-100 ms; RFD 0-200 ms for the least and most affected arm. S4. Ankle joint measurements. These data describes the results of the ankle joint stiffness and ROM measurements. S5. Ankle strength measurements. These data describes the results of the ankle strength measurements in TA. S6. Cognitive and Psychological test. These data describes the data of cardbased CogState computerized tests and test of the spatial location based tasks. S7. Evaluation of personal and social competencies. These data summarize the results of the evaluation of personal and social competencies separated in sections including Overall; Physical abilities; Skills and abilities; Mental well-being; Relation to parents; Relation to others. (DOCX 35 kb) [file 12883_2017_889_MOESM1_ESM.docx]

- **Additional file 1**
- **S1. Functional Tests**

|  | - **Pretest 1** | - **Pretest 2** | - **Post test** |
| --- | --- | --- | --- |
| - **Sit-to-stand** |  |  |  |
| - All (N=17) | - 23.2 (6.3) | - 23.3 (6.2) | - 26.1 (6.3) |
| - CP (N=11) | - 20.5 (5.2) | - 20.2 (4.6) | - **23.5** (5.8)** |
| - TD (N=6) | - 28.3 (523) | - 29.0 (4.5) | - 30.7 (4.5) |
| - **Romberg 30s eyes open** | | | |
| - All (N=17) | - 290 (277)^#^ | - 385 (456) | - 261 (125)^§^ |
| - CP (N=11) | - 363 (331)^#^ | - 486 (535) | - 284 (113)^§^ |
| - TD (N=6) | - 167 (69) | - 202 (174) | - 233 (147) |
| - **Romberg 30s eyes closed** | | | |
| - All (N=17) | - 508 (338)^#^ | - 540 (452) | - 598 (486)^§^ |
| - CP (N=11) | - 556 (427)^#^ | - 613 (550) | - 504 (445)^§^ |
| - TD (N=6) | - 429 (50) | - 408 (119) | - 754 (553) |

- **S1.** Functional tests. Sit-to-stand is number of movement, the Romberg 30 s tests are measured in mm^2^. Data from one participant (^#^ with CP) was lost due to a technical problem. One participant (^§^ with CP) was extremely tired when the balance test was performed on the post test, and almost fell asleep standing. Data from that specific participant is removed from the analyses. **) p<0.01. Data represent means and (SD) from the respective groups.
- **S2. Whole Hand strength and pinch strength**

|  | - **Pretest 1** | | - **Pretest 2** | | - **Post test** | |
| --- | --- | --- | --- | --- | --- | --- |
|  | - **Least aff.** | - **Most aff.** | - **Least aff.** | - **Most aff.** | - **Least aff.** | - **Most aff.** |
| - Hand strength (N) | | | | | | |
| - All (N=17) | - 183 (66) | - 151 (71) | - 178 (71) | - 143 (70) | - 188 (63) | - 154 (68) |
| - CP (N=11) | - 164 (54) | - 129 (61) | - 158 (58) | - 114 (55) | - 167 (51) | - 128 (60) |
| - TD (N=6) | - 216 (79) | - 190 (77) | - 216 (82) | - 195 (68) | - 227 (69) | - 201 (58) |
| - Pinch strength (N) | | | | | | |
| - All (N=17) | - 41.6 (7.8) | - 37.8 (12.4) | - 37.8 (9.3) | - 33.5 (11.2) | - 41.0 (11.1) | - 36.1 (11.8) |
| - CP (N=11) | - 39.4 (5.1) | - 33.9 (13.0) | - 34.8 (6.3) | - 29.4 (8.7) | - 37.5 (7.0) | - 32.2 (8.3) |
| - TD (N=6) | - 45.6 (10.6) | - 44.9 (7.6) | - 43.1 (12.0) | - 40.9 (12.2) | - 47.3 (14.9) | - 43.1 (14.7) |

- **S2:** Hand and pinch strength (in N). No significant improvements were found as a result of the climbing intervention. Data represent means and (SD) from the respective groups. We did not find significant differences in any of the groups when comparing pre2 an post only a trend in the CP group.
- **S3. Pinch rate of force development**

|  | - **Pretest 1** | | - **Pretest 2** | | - **Post test** | |
| --- | --- | --- | --- | --- | --- | --- |
|  | - **Least aff.** | - **Most aff.** | - **Least aff.** | - **Most aff.** | - **Least aff.** | - **Most aff.** |
| - **RFD 0-30ms (N/s)** |  |  |  |  |  |  |
| - All (N=17) | - 55.3 (44.7) | - 49.3 (48.1) | - 62.8 (54.1) | - 47.4 (51.6) | - 77.1 (56.8) | - 54.4 (56.1) |
| - CP (N=11) | - 41.1 (38.4) | - 51.9 (59.0) | - 59.2 (58.6) | - 50.6 (64.4) | - 79.6 (68.6) | - 54.5 (68.6) |
| - TD (N=6) | - 81.2 (47.0) | - 44.6 (20.1) | - 69.3 (49.0) | - 41.6 (13.3) | - 72.6 (29.6) | - 54.2 (25.4) |
| - **RFD 0-50ms (N/s)** |  |  |  |  |  |  |
| - All (N=17) | - 79.3 (60.4) | - 76.3 (60.6) | - 86.3 (63.3) | - 77.2 (64.8) | - 110.7 (65.7) | - 86.7 (69.2) |
| - CP (N=11) | - 59.0 (45.3) | - 78.3 (74.7) | - 79.0 (63.7) | - 80.0 (79.4) | - **109.1 (76.6)*** | - 90.6 (84.8) |
| - TD (N=6) | - 116.5 (70.7) | - 72.5 (24.0) | - 99.7 (66.4) | - 72.1 (28.0) | - 113.6 (45.6) | - 79.6 (29.3) |
| - **RFD 0-100ms (N/s)** |  |  |  |  |  |  |
| - All (N=17) | - 116.0 (74.1) | - 118.8 (69.6) | - 123.8 (67.0) | - 117.6 (70.9) | - 160.5 (60.3) | - 126.9 (66.5) |
| - CP (N=11) | - 89.2 (50.7) | - 111.9 (82.7) | - 110.9 (53.1) | - 109.5 (78.5) | - **154.4 (56.5)**** | - 123.3 (76.8) |
| - TD (N=6) | - 165.2 (89.2) | - 131.5 (39.1) | - 147.5 (87.6) | - 132.3 (57.9) | - 171.6 (70.9) | - 133.4 (47.5) |
| - **RFD 0-200ms (N/s)** |  |  |  |  |  |  |
| - All (N=17) | - 102.9 (49.2) | - 111.2 (54.8) | - 115.3 (43.8) | - 103.0 (49.6) | - 137.9 (37.2) | - 113.5 (45.4) |
| - CP (N=11) | - 85.6 (38.7) | - 102.1 (60.0) | - 105.6 (36.4) | - 91.9 (45.4) | - **133.9 (18.7)*** | - 101.4 (40.5) |
| - TD (N=6) | - 134.7 (53.5) | - 128.0 (43.5) | - 133.2 (53.9) | - 123.4 (54.6) | - 145.4 (60.3) | - 135.6 (49.1) |

- **S3.** Rate of force-development from the most and least affected (CP group) or (non-)dominant hand (TD group). Data represent means and (SD) from the respective groups. We found significant differences in the CP group for the long RFD calculations in the least affected hand when comparing pre2 an post. Statistical significance is a test within a mixed linear model of the effect of climbing intervention specifically tested in each of the two groups. ^o^) p<0.1, *) p<0.05, **) p<0.01, ***) p<0.001.
- **S4. Ankle joint measurements**
- In Table S4 we summarize measurements of ankle joint stiffness and ankle joint range of motion measured by the PSAD device. Unfortunately the PSAD device did not work properly on Pretest2 and we therefore only compare Pretest1 with the Posttest.

|  | - **Pretest 1** | |  | | - **Post test** | |
| --- | --- | --- | --- | --- | --- | --- |
|  | - **Stiffness** | - **ROM** |  |  | - **Stiffness** | - **ROM** |
| - All (N=16”) | - 4.72 (1.79) | - 62.7 (10.38) |  |  | - 4.37 (1.70) | - 67.41 (6.93) |
| - CP (N=11) | - 4.57 (1.86) | - 62.5 (12.00) |  |  | - 4.08 (1.82) | - **67.98* (7.76)** |
| - TD (N=5”) | - 5.05 (1.80) | - 63.12 (6.62) |  |  | - 5.17 (1.18) | - 66.16 (5.16) |

- **S4.** Summary of measurements of passive ankle joint stiffness and ankle joint range of motion”) data from one TDparticipant is missing. Data represent means and (SD) from the respective groups. The CP group showed a significant increase in ROM.
- **S5. Ankle strength measurements**

|  | - **Pretest 1** | - **Posttest** |
| --- | --- | --- |
| - **TA strength (Nm)** |  |  |
| - All (N=17) | - 33.1 (15.7) | - 33.9 (13.2) |
| - CP group (N=11) | - 25.9 (9.5) | - 30.2 (12.0) |
| - TD group (N=6) | - 45.9 (17.5) | - 40.7 (13.8) |

- **S5.** The linear mixed model of TA strength did not reveal a significant difference between the pretest and posttest in the CP-group or the TDgroup in any of the tests.
- **S6. Cognitive and Psychological tests**
- Data from the different tests is presented both in raw form as well as in normalized scores (to 100) based on norm data from previous tests provided by the software company. Statistical tests for each of the different tasks are performed on the normalized scores, which in some tasks combine speed and accuracy into one measure.
- ***S6I. The cardbased CogState computerized tests***

|  | - **Pretest 1** | | | - **Pretest 2** | | | - **Post test** | | |
| --- | --- | --- | --- | --- | --- | --- | --- | --- | --- |
|  |  | | |  | | |  | | |
|  | - Speed (ms) | - Acc. (%) | - Norm | - Speed (ms) | - Acc. (%) | - Norm | - Speed (ms) | - Acc. (%) | - Norm |
| - **DETECT** |  |  |  |  |  |  |  |  |  |
| - All (N=17) | - 423 (125) | - 95.3 (6.1) | - 90.0 (11.7) | - 436 (136) | - 94.3 (6.3) | - 88.6 (12.4) | - 441 (112) | - 92.2 (8.6) | - 87.5 (10.9) |
| - CP (N=11) | - 483 (114) | - 94.4 (7.6) | - 84.1 (9.6) | - 490 (134) | - 92.7 (7.1) | - 83.3 (10.9) | - 486 (109) | - 89.6 (9.6) | - 83.1 (10.0) |
| - TD (N=6) | - 315 (42) | - 97.0 (1.9) | - 100.8 (6.0) | - 336 (70) | - 97.4 (2.3) | - 98.3 (8.5) | - 356 (54) | - 97.0 (3.0) | - 95.7 (7.3) |
| - **IDENT** |  |  |  |  |  |  |  |  |  |
| - All (N=17) | - 644 (117) | - 94.0 (4.3) | - 90.0 (7.1) | - 652 (125) | - 88.7 (8.6) | - 89.7 (8.0) | - 650 (138) | - 91.5 (5.5) | - 90.2 (9.3) |
| - CP (N=11) | - 705 (95) | - 94.1 (5.1) | - 86.1 (5.7) | - 690 (129) | - 88.2 (9.8) | - 87.4 (8.0) | - 692 (146) | - 91.4 (6.2) | - 87.5 (9.6) |
| - TD (N=6) | - 533 (50) | - 93.8 (2.6) | - 97.7 (4.3) | - 582 (90) | - 89.6 (6.8) | - 94.2 (6.3) | - 573 (83) | - 91.6 (4.7) | - 95.3 (6.7) |
| - **1-BACK** |  |  |  |  |  |  |  |  |  |
| - All (N=17) | - 963 (207) | - 81.2 (13.1) |  | - 892 (171) | - 85.5 (11.2) |  | - 889 (227) | - 85.4 (9.4) |  |
| - CP (N=11) | - 1010 (200) | - 78.5 (14.8) |  | - 959 (139) | - 82.3 (9.4) |  | - 962 (214) | - 82.0 (7.9) |  |
| - TD (N=6) | - 877 (208) | - 86.4 (7.9) |  | - 768 (163) | - 91.3 (12.7) |  | - 753 (198) | - 91.7 (9.4) |  |
|  | - Norm | - Norm |  | - Norm | - Norm |  | - Norm | - Norm |  |
|  | - 88.4 (7.8) | - 91.1 (10.7) |  | - 91.1 (7.1) | - 94.9 (10.8) |  | - 91.6 (9.7) | - 94.2 (9.6) |  |
|  | - 86.6 (7.3) | - 88.5 (10.2) |  | - 88.3 (5.3) | - 91.0 (7.5) |  | - 88.5 (9.0) | - 90.3 (6.1) |  |
|  | - 91.7 (8.2) | - 95.7 (11.1) |  | - 96.3 (7.3) | - 102 (12.8) |  | - 97.2 (9.0) | - 101.5 (11.1) |  |

- **S6I.** Summary of card based tasks from the CogState software, these are: DETECT, IDENT and 1-BACK tasks. For the DETECT and IDENT tasks a combined normalized score is provided by the software CogState. For the 1-BACK task two normalized scores, one for speed and one for accuracy are provided. Data represent means and (SD) from the respective groups. We did not find significant differences in any of the groups when comparing pre2 an post.
- ***S6II.Tthe spatial location based tasks***

|  | - **Pretest 1** | | - **Pretest 2** | | - **Post test** | |
| --- | --- | --- | --- | --- | --- | --- |
| - **CHASE** | - Clicks pr. s | | - Clicks pr. s | | - Clicks pr. s |  |
| - All (N=17) | - 1.11 (0.26) | | - 1.21 (0.34) | | - 1.26 (0.26) | |
| - CP (N=11) | - 1.01 (0.25) | | - 1.02 (0.23) | | - 1.15 (0.23) | |
| - TD (N=6) | - 1.26 (0.17) | | - **1.54** (0.23)** | | - 1.48 (0.22) | |
| - **MAZE** | - Errors (#) | - Norm | - Errors (#) | - Norm | - Errors (#) | - Norm |
| - All (N=17) | - 62.8 (22.7) | - 98.5 (8.6) | - 57.9 (14.8) | - 100.2 (5.6) | - 48.6 (15.2) | - 103.9 (5.9) |
| - CP (N=11) | - 71.5 (22.9) | - 95.2 (8.6) | - 59.5 (17.3) | - 99.6 (6.5) | - 50.8 (16.5) | - 103.2 (6.4) |
| - TD (N=6) | - 46.8 (11.4) | - 104.5 (4.5) | - 55.0 (9.3) | - 101.3 (3.7) | - 44.5 (12.6) | - 105.2 (5.1) |
| - **MAZErecall^§^** |  |  |  |  |  |  |
| - All (N=17) | - 9.47 (6.21) |  | - 7.94 (6.04) |  | - 6.41 (3.97) |  |
| - CP (N=11) | - 11.55 (6.86) |  | - 9.36 (7.17) |  | - 7.00 (4.56) |  |
| - TD (N=6) | - 5.67 (1.51) |  | - 5.33 (1.03) |  | - 5.33 (2.58) |  |
| - **PAL** |  |  |  |  |  |  |
| - All (N=17) | - 33.1 (61.7) |  | - 34.9 (43.9) |  | - 23.0 (51.3) |  |
| - CP (N=11) | - 39.4 (74.3) |  | - 45.6 (51.1) |  | - 32.0 (62.9) |  |
| - TD (N=6) | - 21.7 (29.7) |  | - 15.3 (15.8) |  | - 6.5 (3.4) |  |

- **S6II.** Summary of the spatially based tests from CogState.§ The MAZErecall test only consist of a single trial round, and not 5 rounds as the MAZE test, therefore the lower number of errors. **) p<0.01. The TD group show an improvement in the initial CHASE test between pretest 1 and 2. Otherwise we did not find significant differences in any of the groups when comparing pre2 an post. Data represent means and (SD) from the respective groups.
- **S7. Evaluation of personal and social competencies**

|  | - **Pretest 1** | - **Pretest 2** | - **Post test** |
| --- | --- | --- | --- |
| - **Overall** | - Normalized scores | - Normalized scores | - Normalized scores |
| - All (N=17) | - 4.65 (2.23) | - 4.53 (2.24) | - 5.30 (2.42) |
| - CP (N=11) | - 4.00 (1.94) | - 4.00 (2.05) | - 4.63 (2.41) |
| - TD (N=6) | - 5.83 (2.40) | - 5.5 (2.42) | - 6.50 (2.07) |
| - **Physical abilites** | |  |  |
| - All (N=17) | - 4.41 (2.03) | - 5.06 (2.77) | - 4.94 (2.82) |
| - CP (N=11) | - 3.91 (1.64) | - 4.64 (2.73) | - 4.45 (2.73) |
| - TD (N=6) | - 5.33 (2.40) | - 5.83 (2.93) | - 5.83 (2.99) |
| - **Skills and abilites** | |  |  |
| - All (N=17) | - 4.53 (2.24) | - 4.47 (2.26) | - 4.88 (2.32) |
| - CP (N=11) | - 4.36 (2.20) | - 4.54 (2.25) | - 4.45 (2.38) |
| - TD (N=6) | - 4.83 (2.48) | - 4.33 (2.50) | - 5.67 (2.16) |
| - **Mental well-being** | |  |  |
| - All (N=17) | - 5.24 (2.46) | - 4.71 (2.17) | - 5.52 (2.47) |
| - CP (N=11) | - 4.72 (2.33) | - 4.09 (2.21) | - 4.91 (2.59) |
| - TD (N=6) | - 6.17 (2.63) | - 5.83 (1.72) | - 6.67 (1.96) |
| - **Relation to parents** | |  |  |
| - All (N=17) | - 5.06 (2.38) | - 4.65 (2.69) | - 5.11 (2.47) |
| - CP (N=11) | - 4.00 (1.89) | - 3.18 (1.89) | - 4.18 (2.36) |
| - TD (N=6) | - 7.00 (2.00) | - 7.33 (1.63) | - 6.83 (1.72) |
| - **Relation to others** | |  |  |
| - All (N=17) | - 5.18 (2.03) | - 5.59 (2.31) | - 5.88 (2.23) |
| - CP (N=11) | - 4.36 (1.86) | - 4.45 (2.06) | - 5.27 (2.37) |
| - TD (N=6) | - 6.67 (1.51) | - 7.67 (0.82) | - 7.00 (1.55) |

- **S7.** Summary of the self-reported questionnaire “Sådan er jeg” All results are normalized (range 0-9) to a reference data set obtained from approximately 400 school children in a similar age range. Data represent means and (SD) from the respective groups.
